# Effect of Electroacupuncture on Insomnia in Patients With Depression: A Randomized Clinical Trial

**DOI:** 10.1001/jamanetworkopen.2022.20563

**Published:** 2022-07-07

**Authors:** Xuan Yin, Wei Li, Tingting Liang, Bing Lu, Hongyu Yue, Shanshan Li, Victor W. Zhong, Wei Zhang, Xia Li, Shuang Zhou, Yiqun Mi, Huangan Wu, Shifen Xu

**Affiliations:** 1 Shanghai Municipal Hospital of Traditional Chinese Medicine, Shanghai University of Traditional Chinese Medicine, Shanghai, China; 2 School of Basic Medical Sciences, Shanghai Medical College, Fudan University, Shanghai, China; 3 Jiading Branch of Shanghai General Hospital, Shanghai Jiao Tong University School of Medicine, Shanghai, China; 4 Department of Acupuncture and Moxibustion, Huadong Hospital, Fudan University, Shanghai, China; 5 School of Public Health, Shanghai Jiao Tong University School of Medicine, Shanghai, China; 6 School of Public Health, Fudan University, Shanghai, China; 7 Shanghai Mental Health Center, Shanghai Jiao Tong University School of Medicine, Shanghai, China; 8 Department of Traditional Chinese Medicine, Changhai Hospital, Navy Medical University, Shanghai, China; 9 Shanghai Research Institute of Acupuncture and Meridian, Shanghai University of Traditional Chinese Medicine, Shanghai, China

## Abstract

**Importance:**

Electroacupuncture (EA) is a widely recognized therapy for depression and sleep disorders in clinical practice, but its efficacy in the treatment of comorbid insomnia and depression remains uncertain.

**Objective:**

To assess the efficacy and safety of EA as an alternative therapy in improving sleep quality and mental state for patients with insomnia and depression.

**Design, Setting, and Participants:**

A 32-week patient- and assessor-blinded, randomized, sham-controlled clinical trial (8-week intervention plus 24-week observational follow-up) was conducted from September 1, 2016, to July 30, 2019, at 3 tertiary hospitals in Shanghai, China. Patients were randomized to receive EA treatment and standard care, sham acupuncture (SA) treatment and standard care, or standard care only as control. Patients were 18 to 70 years of age, had insomnia, and met the criteria for depression as classified in the *Diagnostic and Statistical Manual of Mental Disorders (Fifth Edition)*. Data were analyzed from May 4 to September 13, 2020.

**Interventions:**

All patients in the 3 groups were provided with standard care guided by psychiatrists. Patients in the EA and SA groups received real or sham acupuncture treatment, 3 sessions per week for 8 weeks, for a total of 24 sessions.

**Main Outcomes and Measures:**

The primary outcome was change in Pittsburgh Sleep Quality Index (PSQI) from baseline to week 8. Secondary outcomes included PSQI at 12, 20, and 32 weeks of follow-up; sleep parameters recorded in actigraphy; Insomnia Severity Index; 17-item Hamilton Depression Rating Scale score; and Self-rating Anxiety Scale score.

**Results:**

Among the 270 patients (194 women [71.9%] and 76 men [28.1%]; mean [SD] age, 50.3 [14.2] years) included in the intention-to-treat analysis, 247 (91.5%) completed all outcome measurements at week 32, and 23 (8.5%) dropped out of the trial. The mean difference in PSQI from baseline to week 8 within the EA group was −6.2 (95% CI, −6.9 to −5.6). At week 8, the difference in PSQI score was −3.6 (95% CI, −4.4 to −2.8; *P* < .001) between the EA and SA groups and −5.1 (95% CI, −6.0 to −4.2; *P* < .001) between the EA and control groups. The efficacy of EA in treating insomnia was sustained during the 24-week postintervention follow-up. Significant improvement in the 17-item Hamilton Depression Rating Scale (−10.7 [95% CI, −11.8 to −9.7]), Insomnia Severity Index (−7.6 [95% CI, −8.5 to −6.7]), and Self-rating Anxiety Scale (−2.9 [95% CI, −4.1 to −1.7]) scores and the total sleep time recorded in the actigraphy (29.1 [95% CI, 21.5-36.7] minutes) was observed in the EA group during the 8-week intervention period (*P* < .001 for all). No between-group differences were found in the frequency of sleep awakenings. No serious adverse events were reported.

**Conclusions and Relevance:**

In this randomized clinical trial of EA treatment for insomnia in patients with depression, quality of sleep improved significantly in the EA group compared with the SA or control group at week 8 and was sustained at week 32.

**Trial Registration:**

ClinicalTrials.gov Identifier: NCT03122080

## Key Points

**Question:**

What is the efficacy of electroacupuncture in treating insomnia among patients with depression?

**Findings:**

In this randomized clinical trial including 270 patients with insomnia and depression who underwent 8 weeks of electroacupuncture with a 24-week observational follow-up, electroacupuncture with standard care significantly improved patients’ quality of sleep compared with sham acupuncture with standard care and standard care alone.

**Meaning:**

This study found that electroacupuncture with standard care significantly alleviated insomnia among patients with depression.

## Teaser

This randomized clinical trial assesses the efficacy and safety of electroacupuncture as a treatment for insomnia among patients with depression and whether it is superior to sham acupuncture or standard care.

## Introduction

Depressive disorders, which are characterized by depressed mood, loss of interest, and reduced energy leading to diminished activity, are one of the most commonly diagnosed psychiatric disorders, affecting more than 264 million people worldwide.^1^ More than 50 million people in China (3.6% of the population) experience depressive disorders each year.^2^ Sleep disturbance is the prominent symptom in patients with depression, and in China 64.6% of them reported insomnia.^3^ Depression and sleep issues have a bidirectional relationship. Poor sleep quality contributes to the development of depression, and having depression makes a person more likely to develop sleep issues.^4,5^ Patients with co-occurring depression and sleep disorders have a higher risk of suicide and severe impairment in social functioning than patients who have depression alone.^6,7^ These co-occurring disorders are difficult to treat and confer a greater risk of relapse and recurrence of depression.^8,9^

Cognitive behavioral therapy for insomnia is an effective therapy that focuses on managing chronic insomnia.^10,11^ However, the cultural difference and limited medical resources compromise its large-scale application in China.^12^ Therefore, rates of antidepressant prescriptions continue to increase in Chinese populations, which raises concerns.^13^ Some antidepressants with activating effects may deteriorate sleep during short-term treatment. Others with sedative properties rapidly improve sleep but may cause oversedative problems in the long term and may even worsen patient outcomes.^14,15^

Many patients with depression in China have not received adequate treatment yet, and many are nonresponsive to current interventions.^16^ The high prevalence of untreated depression suggests a need to promote accessible and easily implementable interventions. Acupuncture has been regarded as a potentially effective drug-free approach for helping to treat mental illness and sleep disorders.^17,18,19^ A previous study^20^ demonstrated that acupuncture effectively improves sleep efficacy and prolongs total sleep time in patients with primary insomnia. A pilot study on electroacupuncture (EA) for the treatment of depression-related insomnia^21^ found that patients experienced significant improvement in sleep quality after receiving EA treatment during a 12-week study period. However, the small sample size may cause uncertainty and nonrepeatability, and the effect duration of EA treatment also remains unknown.

This 32-week multicentered, randomized clinical trial was conducted to assess the efficacy and safety of EA in treating insomnia among patients with depression. Furthermore, we investigated whether EA was superior to sham acupuncture (SA) or standard care in treating patients experiencing comorbid insomnia and depression.

## Methods

### Study Population and Protocol

This multicenter, randomized, sham-controlled clinical trial was conducted from September 1, 2016, to July 30, 2019, at the Shanghai Municipal Hospital of Traditional Chinese Medicine, Shanghai Mental Health Center, and Changhai Hospital in Shanghai, China. The trial design had been published previously,^22^ and the protocol was approved by the ethics committee of Shanghai Municipal Hospital of Traditional Chinese Medicine and monitored by an independent data and safety monitoring board (Supplement 1). All patients provided written informed consent before participation. We followed the Consolidated Standards of Reporting Trials (CONSORT) reporting guideline and the Standards for Reporting Interventions in Clinical Trials of Acupuncture (STRICTA) guideline^23^ for the designing and reporting of this trial.

The study population included patients with insomnia who fulfilled the following criteria: men or women aged 18 to 70 years whose Pittsburgh Sleep Quality Index (PSQI) was greater than 7 (range, 0-21, with higher scores indicating worse quality of sleep and more sleep disorders), who met *Diagnostic and Statistical Manual of Mental Disorders (Fifth Edition)* criteria for depression, who had a 17-item Hamilton Depression Rating Scale (HDRS-17) score of 20 to 35 (range, 0-52, with higher scores indicating higher depression levels), and who were consistently with or without regular use of antidepressants for 4 weeks before the study began. The main exclusion criteria consisted of secondary depressive disorders caused by organic diseases, medicine, or psychotic disorders; being in a depressive episode of bipolar disorder or experiencing dysthymia, reactive depression, and depressive syndrome caused by other diseases; severe diseases of cardiovascular or hematopoietic systems or poor liver or kidney function; a history of alcohol abuse or drug dependence; pregnancy or lactation among women; or having received acupuncture treatment within the past 1 year.

### Randomization and Blinding

Eligible patients were randomized to receive EA, SA, and/or standard care with a 1:1:1 ratio by an online central randomization system (Shanghai BioGuider Medicinal Technology). Independent staff established the data analysis system for the Electronic Data Capture software, version 5.0 (Shanghai BioGuider Medicinal Technology), and prepared the randomization database.

Patients in the EA and SA groups were kept blinded to their group assignment. They were treated in a closed unit, wearing eye masks during each session. The success of the blinding method was validated at the end of the 8-week intervention by the Bang blinding index.^24^ Except for the acupuncturists, other researchers—including the statisticians, outcome assessors, and data analysts—were all blinded to the group assignments. The acupuncturists were not involved in the outcome assessment or data analysis.

### Study Procedures

After central randomization, patients underwent a baseline assessment of sleep status using the PSQI, the Insomnia Severity Index (ISI; range, 0-28, with higher scores indicating worse quality of sleep), and actigraphy and assessment of their depression status using the HDRS-17. Anxiety was assessed by the Self-rating Anxiety Scale (range, 20-80, with higher scores indicating worse anxiety), and the dose of antidepressants (if taken) was recorded. The assessments of patients’ sleep and mental condition using the PSQI, ISI, HDRS-17, Self-rating Anxiety Scale, and actigraphy were given in the middle (week 4) and at the end (week 8) of the treatment. The PSQI and HDRS-17 were also assessed during the observational follow-up period (weeks 12, 20, and 32). Adverse events were monitored throughout the trial and recorded at all assessment time points.

### Interventions

Patients in the EA or SA groups received a 30-minute treatment 3 times per week (usually every other day except Sunday) for 8 consecutive weeks. All treatments were performed by licensed acupuncturists with at least 5 years of clinical experience. A total of 6 acupuncturists (2 at each center; including X.Y. and S.Z.) performed EA and SA, and they received standardized training on the intervention method before the trial. The regular acupuncture method was applied at the Baihui (GV20), Shenting (GV24), Yintang (GV29), Anmian (EX-HN22), Shenmen (HT7), Neiguan (PC6), and SanYinjiao (SP6) acupuncture points, with 0.25 × 25-mm and 0.30 × 40-mm real needles (Wuxi Jiajian Medical Device Co, Ltd), or 0.30 × 30-mm sham needles (Streitberger sham device [Asia-med GmbH]).

For patients in the EA group, rotating or lifting-thrusting manipulation was applied for deqi sensation after needle insertion. The 2 electrodes of the electrostimulator (CMNS6-1 [Wuxi Jiajian Medical Device Co, Ltd]) were connected to the needles at GV20 and GV29, delivering a continuous wave based on the patient’s tolerance. Patients in the SA group felt a pricking sensation when the blunt needle tip touched the skin, but without needle insertion. All indicators of the nearby electrostimulator were set to 0, with the light switched on. The selection and location of acupuncture points are shown in the eFigure in Supplement 2. Standard care (also known as treatment as usual or routine care) was used in the control group.^25^ Patients receiving standard care were recommended by the researchers to get regular exercise, eat a healthy diet, and manage their stress level during the trial. They were asked to keep the regular administration of antidepressants, sedatives, or hypnotics as well.^16^ Psychiatrists in the Shanghai Mental Health Center (including X.L.) guided all patients’ standard care treatment and provided professional advice when a patient’s condition changed.

### Measures

The primary outcome was change in PSQI score at the end of the intervention (week 8). The PSQI is a widely used questionnaire to assess one’s sleep quality for the past month, with 7 components for 7 specific features of sleep. Secondary outcomes included HDRS-17 and Self-rating Anxiety Scale scores, which provided a subjective assessment of patients’ mental health state, and the actigraphy data, consisting of sleep efficiency, times of sleep awakenings, and total sleep time, which provided a relatively objective assessment of patients’ sleep status. The ISI offered complementary information for the severity of both nighttime and daytime components of insomnia. Doses of sedatives were also a secondary outcome.

Any adverse event, such as unfavorable or unintended signs, symptoms, or diseases, related to the acupuncture treatment or the administration of antidepressants was reported by patients and acupuncturists. Severe adverse events had to be reported to the principal investigator and the data and safety monitoring board within 24 hours after their occurrence.

### Statistical Analysis

Data were analyzed from May 4 to September 13, 2020. The sample size calculation was based on a previous study^22^ conducted in 2016 (n = 90), showing that the mean (SD) PSQI scores in the EA group were 9.8 (3.1) and in the SA group were 13.9 (3.2) after an 8-week intervention. Assuming the superior effect as 1.5 of the difference of PSQI, with an α value of .025, a β value of 0.1, and a 10% dropout rate, a sample size of 30 for each group was needed at each site. Therefore, a total of 270 participants were needed for this trial.

All statistical analyses were based on the intention-to-treat principle of all randomly assigned patients, with significance level indicated by 1-sided *P* < .025. The descriptive analysis was used for the baseline characteristics of the patients in each group. For the primary outcome, the PSQI was assessed by the linear mixed-effects model with the interaction effects of time and group. Missing data were replaced according to the principle of the last observation carried forward. The HDRS-17 was assessed by the mixed model. Analysis of covariance with baseline adjustment was used for other indicators, including PSQI, ISI, Self-rating Anxiety Scale, sleep efficiency, sleep awakenings, and total sleep time. A Bonferroni correction was used to account for multiple comparisons. All statistical analyses were conducted with SAS Deployment Wizard, version 9.4 (SAS Institute Inc).

## Results

### Patient Characteristics

**Figure 1.**
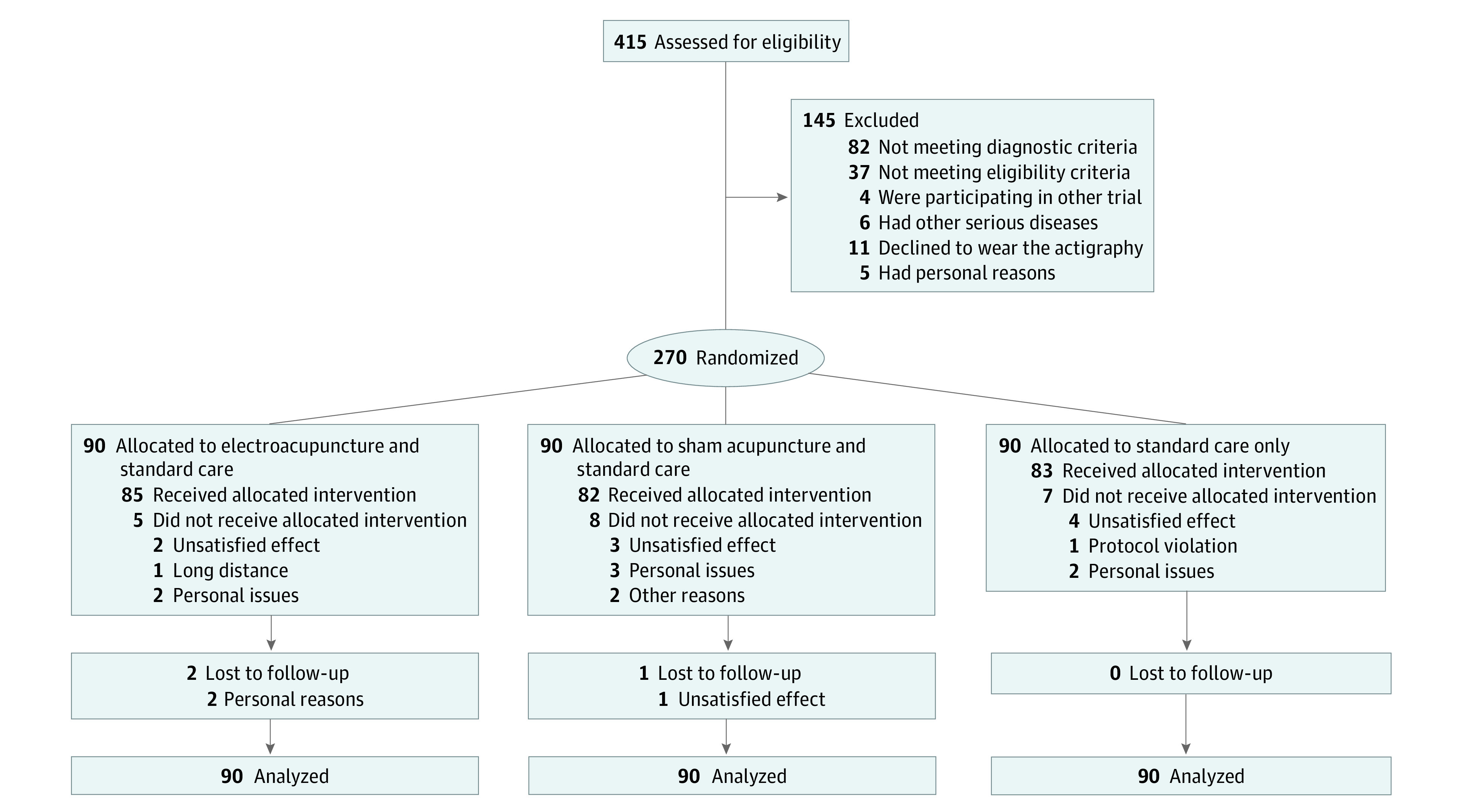
Study Flowchart

A total of 415 patients with comorbid insomnia and depression were screened between September 1, 2016, and October 1, 2018, in 3 hospitals, and 270 eligible patients were enrolled, including 194 women (71.9%) and 76 men (28.1%) with a mean (SD) age of 50.3 (14.2) years (Figure 1). The demographic and clinical characteristics of the patients are provided in Table 1. Follow-up was completed on July 30, 2019. A total of 247 patients (91.5%) completed all outcome measurements at week 32, and 23 patients (8.5%) dropped out of the study owing to time commitment, personal issues, or unsatisfying effects (7 in the EA group, 9 in the SA group, and 7 in the control group; 20 during the intervention period and 3 during the 24-week follow-up period). Of the patients who completed the 8-week intervention, 83 were in the EA group, 81 were in the SA group, and 83 were in the control group.

**Table 1.** Baseline Characteristics of the Intention-to-Treat Population

Characteristic	Study group^a^		
	EA (n = 90)	SA (n = 90)	Control (n = 90)
Age, mean (SD), y	50.9 (14.0)	50.5 (14.0)	49.6 (14.9)
Duration of depression, mean (SD), wk	447.9 (417.8)	355.1 (397.1)	389.5 (436.7)
Sex			
Men	27 (30.0)	30 (33.3)	19 (21.1)
Women	63 (70.0)	60 (66.7)	71 (78.9)
Marital status			
Married	73 (81.1)	71 (78.9)	70 (77.8)
Not married	11 (12.2)	14 (15.5)	15 (16.7)
Divorced	3 (3.3)	2 (2.2)	3 (3.3)
Widowed	3 (3.3)	3 (3.3)	2 (2.2)
Educational attainment			
Graduate	7 (7.8)	4 (4.4)	8 (8.9)
Undergraduate	43 (47.8)	49 (54.4)	36 (40.0)
High school and below	40 (44.4)	37 (41.1)	46 (51.1)
Employment status			
Employed	51 (56.7)	54 (60.0)	55 (61.1)
Retired	39 (43.3)	36 (40.0)	35 (38.9)
Previous treatment in past year			
Yes	70 (77.8)	74 (82.2)	68 (75.5)
No	20 (22.2)	16 (17.8)	22 (24.4)
Coffee or tea drinking habit			
Yes	9 (10.0)	11 (12.2)	8 (8.9)
No	81 (90.0)	79 (87.8)	82 (91.1)
Other chronic diseases			
Yes	23 (25.5)	36 (40.0)	33 (36.7)
No	67 (74.4)	54 (60.0)	57 (63.3)
Sedative medicine			
Yes	15 (16.7)	18 (20.0)	15 (16.7)
No	75 (83.3)	72 (80.0)	75 (83.3)
Questionnaire scores, mean (SD)			
PSQI^b^	15.1 (2.9)	14.7 (3.1)	15.2 (2.9)
HDRS-17^c^	24.0 (3.2)	23.6 (3.1)	23.8 (3.3)
ISI^d^	19.3 (3.6)	19.5 (4.0)	18.7 (3.6)
Self-rating Anxiety Scale^e^	49.5 (6.9)	49.8 (6.9)	49.9 (7.4)
Sleep, mean (SD)			
Efficiency, %	78.5 (9.8)	80.8 (10.8)	79.4 (10.0)
Total time, min	375.9 (47.1)	397.0 (59.5)	386.4 (64.2)
Awakening, No. of times	21.8 (9.3)	20.3 (8.7)	20.0 (9.7)

Abbreviations: EA, electroacupuncture; HDRS-17, 17-item Hamilton Rating Scale for Depression; ISI, Insomnia Severity Index; PSQI, Pittsburgh Sleep Quality Index; SA, sham acupuncture.

^a^ Unless indicated otherwise, data are expressed as No. (%) of patients. Percentages have been rounded and may not total 100.

^b^ Scores range from 0 to 21, with higher scores indicating worse quality of sleep and more sleep disorders.

^c^ Scores range from 0 to 52, with higher scores indicating higher depression levels.

^d^ Scores range from 0 to 28, with higher scores indicating worse quality of sleep.

^e^ Scores range from 20 to 80, with higher scores indicating worse anxiety.

### Efficacy

**Figure 2.**
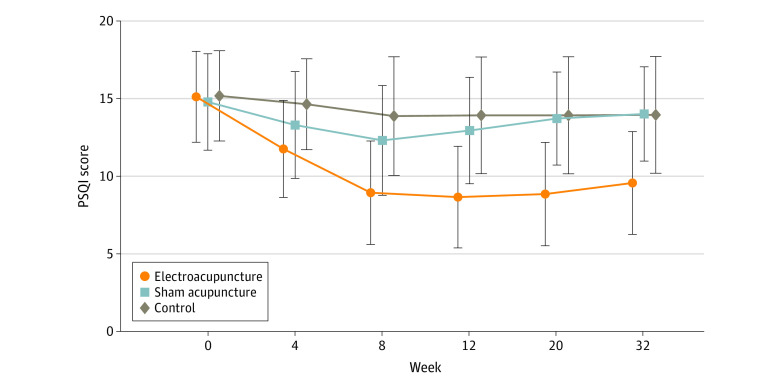
Changes in Pittsburgh Sleep Quality Index (PSQI) Scores Among Groups Over Time Higher PSQI scores indicate worse quality of sleep and more sleep disorders.

Table 2 shows the PSQI scores of all 3 groups; the results were analyzed to reveal changes from baseline (week 0) to 24-week follow-up (week 32). At the 8-week posttreatment assessment (primary end point), patients who had received EA treatment had a mean reduction of 6.2 (95% CI, −6.9 to −5.6) points in the PSQI score from baseline. The EA group reported a significant between-group difference in PSQI score of −3.6 points (95% CI, −4.4 to −2.8 points; *P* < .001) compared with the SA group and −5.1 (95% CI, −6.0 to −4.2; *P* < .001) points compared with the control group after the 8-week intervention period. The trends of PSQI scores in the 3 groups are shown in Figure 2.

**Table 2.** Pittsburgh Sleep Quality Index (PSQI) Scores Among Study Participants

Outcome assessment	Mean change in PSQI score from baseline (95% CI)^a^			EA vs SA groups		EA vs control groups	
	EA group	SA group	Control group	Difference (95% CI)	*P* value^b^	Difference (95% CI)	*P* value^b^
Week 4	−3.4 (−4.0 to −2.8)	−1.5 (−1.9 to −1.1)	−0.6 (−1.0 to −0.2)	−1.8 (−2.5 to −1.1)	<.001	−2.9 (−3.5 to −2.2)	<.001
Week 8	−6.2 (−6.9 to-5.6)	−2.5 (−3.1 to −1.9)	−1.1 (−1.8 to −0.5)	−3.6 (−4.4 to −2.8)	<.001	−5.1 (−6.0 to −4.2)	<.001
Week 12	−6.5 (−7.2 to −5.8)	−1.8 (−2.3 to −1.3)	−1.1 (−1.7 to −0.5)	−4.5 (−5.4 to −3.7)	<.001	−5.5 (−6.4 to −4.6)	<.001
Week 20	−6.3 (−7.0 to −5.6)	−1.1 (−1.5 to −0.7)	−0.9 (−1.5 to −0.2)	−5.1 (−5.9 to −4.4)	<.001	−5.5 (−6.4 to −4.7)	<.001
Week 32	−5.6 (−6.3 to −4.9)	−0.8 (−1.2 to −0.4)	−0.7 (−1.3 to −0.1)	−4.7 (−5.4 to −3.9)	<.001	−5.0 (−5.8 to −4.1)	<.001

Abbreviations: EA, electroacupuncture; SA, sham acupuncture.

^a^ Scores range from 0 to 21, with higher scores indicating worse quality of sleep and more sleep disorders.

^b^ Compared using Bonferroni correction.

Table 3 summarizes the results of secondary outcomes. Compared with the SA group and the control group, patients in the EA group had a significantly greater reduction in PSQI at weeks 4 (−1.8 [95% CI, −2.5 to −1.1] and −2.9 [95% CI, −3.5 to −2.2], respectively), 12 (−4.5 [95% CI, −5.4 to −3.7] and −5.5 [95% CI, −6.4 to −4.6], respectively), 20 (−5.1 [95% CI, −5.9 to −4.4] and −5.5 [95% CI, −6.4 to −4.7], respectively), and 32 (−4.7 [95% CI, −5.4 to −3.9] and −5.0 [95% CI, −5.8 to −4.1], respectively) (*P* < .001). Significant improvements in 17-item Hamilton Depression Rating Scale (−10.7 [95% CI, −11.8 to −9.7]), Insomnia Severity Index (−7.6 [95% CI, −8.5 to −6.7]), and Self-rating Anxiety Scale (−2.9 [95% CI, −4.1 to −1.7] scores and total sleep time recorded in the actigraphy (29.1 [95% CI, 21.5-36.7] minutes) were observed in the EA group during the 8-week intervention period (*P* < .001 for all). During the 8-week treatment and the 24-week follow-up period, the EA group had a significantly lower depression score of HDRS-17 than the SA group (−5.5 [95% CI, −6.8 to −4.3] and −5.8 [95% CI, −6.8 to −4.7], respectively) and control group (−8.8 [95% CI, −10.1 to −7.4] and −5.8 [95% CI, −7.1 to −4.5], respectively) (*P* < .001 for all). Patients in the EA group had a 4.2% (95% CI, 2.6%-5.8%) higher sleep efficiency than those in the SA group at week 8 (*P* < .001), whereas no significant difference was identified at week 4 (1.8% (95% CI, −0.2% to 3.7%]; *P* = .15). During the treatment period, the EA group had lower scores in the ISI and Self-rating Anxiety Scale and longer total sleep time recorded in the actigraphy than the control group; similar results were found when compared with the SA group except for the Self-rating Anxiety Scale score at week 8.

**Table 3.** Patients’ Mental State and Sleep Quality as Secondary Outcomes

Outcome	Mean change from baseline (95% CI)			EA vs SA groups		EA vs control groups	
	EA group	SA group	Control group	Difference (95% CI)	*P* value^a^	Difference (95% CI)	*P* value^a^
HDRS-17^b^							
Week 4	−7.0 (−8.0 to −6.0)	−3.7 (−4.4 to −2.9)	−1.3 (−2.1 to −0.4)	−3.1 (−4.3 to −2.0)	<.001	−5.7 (−6.9 to −4.5)	<.001
Week 8	−10.7 (−11.8 to −9.7)	−5.0 (−5.8 to −4.1)	−1.9 (−2.9 to −0.8)	−5.5 (−6.8 to −4.3)	<.001	−8.8 (−10.1 to −7.4)	<.001
Week 12	−10.9 (−11.9 to −9.8)	−4.2 (−5.1 to −3.4)	−2.2 (−3.3 to −1.1)	−6.4 (−7.6 to −5.2)	<.001	−8.6 (−10.0 to −7.2)	<.001
Week 20	−9.2 (−10.2 to −8.2)	−3.1 (−3.9 to −2.3)	−1.9 (−3.0 to −0.8)	−5.9 (−7.0 to −4.8)	<.001	−7.3 (−8.6 to −5.9)	<.001
Week 32	−7.7 (−8.7 to −6.8)	−1.8 (−2.6 to −1.0)	−1.9 (−3.0 to −0.8)	−5.8 (−6.8 to −4.7)	<.001	−5.8 (−7.1 to −4.5)	<.001
ISI^c^							
Week 4	−4.5 (−5.2 to −3.8)	−1.9 (−2.4 to −1.4)	−0.7 (−1.3 to −0.1)	−2.6 (−3.4 to −1.8)	<.001	−3.7 (−4.6 to −2.8)	<.001
Week 8	−7.6 (−8.5 to −6.7)	−3.3 (−4.1 to −2.6)	−1.4 (−2.2 to −0.7)	−4.3 (−5.4 to −3.3)	<.001	−6.0 (−7.1 to −4.8)	<.001
Self-rating Anxiety Scale^d^							
Week 4	−2.0 (−3.0 to −1.1)	−0.4 (−1.1 to 0.2)	0.26 (−0.6 to 1.1)	−1.7 (−2.6 to −0.7)	<.001	−2.4 (−3.5 to −1.3)	<.001
Week 8	−2.9 (−4.1 to −1.7)	−1.4 (−2.5 to −0.3)	0.14 (−0.9 to 1.2)	−1.6 (−3.0 to −0.1)	.07	−3.2 (−4.6 to −1.7)	<.001
Sleep efficiency							
Week 4	80.7 (0.3 to 4.0)	80.5 (−1.2 to 0.8)	79.1 (−1.4 to 0.4)	1.8 (−0.2 to 3.7)	.15	2.4 (0.5 to 4.4)	.03
Week 8	84.4 (4.2 to 7.2)	81.6 (−0.4 to 1.9)	79.8 (−0.8 to 0.9)	4.2 (2.6 to 5.8)	<.001	5.4 (3.9 to 6.9)	<.001
Total sleep time, min							
Week 4	14.2 (7.9 to 20.5)	−6.0 (−13.4 to 1.4)	−6.4 (−12.5 to −0.4)	13.9 (5.5 to 22.4)	.004	17.3 (9.9 to 24.7)	<.001
Week 8	29.1 (21.5 to 36.7)	−7.0 (−17.9 to 3.9)	−3.3 (−14.6 to 8.1)	27.2 (15.8 to 38.7)	<.001	26.6 (15.8 to 37.4)	<.001
No. of sleep awakenings							
Week 4	−0.1 (−1.3 to 1.0)	0.5 (−0.7 to 1.6)	0.8 (−0.2 to 1.8)	−0.2 (−1.7 to 1.3)	.76	−0.5 (−1.9 to 0.9)	.46
Week 8	−0.8 (−2.9 to 1.3)	0.6 (−0.9 to 2.0)	0.9 (0.0 to 1.7)	−1.0 (−3.4 to 1.5)	.44	−1.4 (−3.6 to 0.9)	.24

Abbreviations: EA, electroacupuncture; HDRS-17, 17-item Hamilton Rating Scale for Depression; ISI, Insomnia Severity Index; SA, sham acupuncture.

^a^ Calculated using Bonferroni correction.

^b^ Scores range from 0 to 52, with higher scores indicating higher depression levels.

^c^ Scores range from 0 to 28, with higher scores indicating worse quality of sleep.

^d^ Scores range from 20 to 80, with higher scores indicating worse anxiety.

Results for each of the 7 components of PSQI in the 3 groups during the intervention period are provided in eTable 1 in Supplement 2. Although no significant between-group differences were identified in the habitual sleep efficiency at week 4, significant differences were found in all other components, including subjective sleep quality (EA vs SA, −0.7 [95% CI, −0.8 to −0.5]; EA vs control, −1.0 [95% CI, −1.2 to −0.8]), sleep latency (EA vs SA, −0.4 [95% CI, −0.6 to −0.1]; EA vs control, −0.5 [95% CI, −0.7 to −0.3]), sleep duration (EA vs SA, −0.5 [95% CI, −0.8 to −0.3]; EA vs control, −0.7 [95% CI, −0.9 to −0.5), and sleep disturbance (EA vs SA, −0.3 [95% CI, −0.5 to −0.2]; EA vs control, −0.4 [95% CI, −0.6 to −0.3]) at week 8 (*P* ≤ .004). However, there were no significant between-group differences in sleep awakenings at weeks 4 and 8.

The changes of patients’ dose of sedatives during the intervention are listed in eTable 2 in Supplement 2. Only 1 patient in the EA group increased his or her sedative dosagae, whereas more patients were recorded to adjust their dosage in other groups.

### Safety

Acupuncture-related adverse events occurred in 7 patients (7.8%) in the EA group and 4 patients (4.4%) in the SA group. In 1 case (1.1%), a patient in the control group had a headache during the observation period. No significant difference was found among groups for the proportion of patients with adverse events (*P* = .10). The most commonly reported acupuncture-related adverse events were hematoma and local pain. No patients had severe adverse events in the trial (eTable 3 in Supplement 2).

### Blinding

The result of the blinding assessment (eTable 4 in Supplement 2) showed that 56 patients (62.2%) in the SA group guessed wrongly about their group assignment (Bang blinding index, −0.4 [95% CI, −0.6 to −0.3]), whereas 15 (16.7%) in the EA group also guessed wrongly (Bang blinding index, 0.5 [95% CI, 0.4-0.7]). This indicated a relatively higher degree of blinding in the SA group.

## Discussion

Our multicenter randomized sham-controlled clinical trial found that compared with SA and standard care, EA treatment resulted in a significantly lower PSQI score by the end of the intervention period at week 8, and this improvement in sleep quality persisted during the 24-week observational follow-up. Patients in the EA group also had a greater reduction of severity of insomnia, depressive mood, and anxiety symptoms at the end of the intervention. There were no severe adverse events reported during the trial, indicating that EA may be a safe treatment for patients with comorbid depression and insomnia.

At the end of an 8-week intervention, a significant 6.2-point decrease in PSQI score was observed in the EA treatment group. At the end of the observation period, the EA with control group had a significant 3.6-point lower PSQI score and a significant 5.5-point lower HDRS-17 score than the SA with standard care group as well as a 5.1-point lower PSQI score and an 8.8-point lower HDRS-17 score than standard care alone. Previous studies^26,27^ have suggested that the minimum clinically important difference is more than 3 points, and patients who have improved their PSQI score of 4.4 from baseline to 6 months of follow-up have shown a clinically significant increase in their health status.^28^ In the present trial, EA treatment with standard care was clinically beneficial and superior to SA and/or standard care for treating insomnia and helped patients to improve their mental health status. Previous work has shown that it can be more challenging to treat insomnia among patients with a longer duration of depression.^29^ In the present study, the mean duration of depression in the EA group was longer than that in the other 2 groups at baseline, but a significant improvement in sleep quality was still observed in the EA group.

Acupuncture has been used to treat depressive disorders and sleep disturbances in China for thousands of years. Previous studies^30,31^ have shown that acupuncture has little effect on treating depression combined with insomnia. However, such findings were hampered by flaws in study design, including short treatment duration and different acupoint prescriptions, as well as variable acupuncturists’ skills.^32^ In the present trial, the effects of the EA treatment on objective sleep maintenance and architecture were clearly observed by the actigraphy. Use of EA yielded significant improvements in sleep efficiency and total sleep time and a decreasing trend in the number of sleep awakenings. This trial included a longer study period, experienced acupuncturists, and a selection of important acupoints on the Governor vessel. The Governor vessel governs all yang meridians and helps regulate the balance of yin-yang in the body, which is closely related to brain functions.^33,34^ It can calm nerves, relax patients, and alleviate their depressive symptoms.^35^ To our knowledge, there have been no similar large-scale multicenter randomized clinical trials studying the effects of EA on treating comorbid depression and insomnia. This strictly designed rigorously conducted trial provides important clinical evidence about the role and value of EA as an alternative therapy for treating insomnia and depressive moods.

Real acupuncture induces both specific and nonspecific effects, whereas placebo acupuncture produces only nonspecific effects. An ideal placebo to minimize the nonspecific effects should be noninvasive. We applied the non–needle insertion Streitberger sham device in this trial. We also evaluated the blinding method to test whether patients knew about the assignment at the end of the intervention. Results of the Bang blinding index in the SA group indicated patients guessed in an opposite direction. Patients in the SA group believed that they took the real acupuncture treatment, and thus the blinding in the SA group was successful. However, the result also suggested that patients receiving the EA treatment had guessed correctly about their group assignment, which may to some extent increase their treatment expectation and inevitably increase placebo response.

### Limitations

Our study has several limitations. First, acupuncturists were not blinded to the group assignment owing to the treatment procedure. To minimize potential bias due to the single-blinding method, 2 independent acupuncturists at each site were assigned to perform the real and sham EA treatment and adhere to the principle of task separation. Second, the blinding assessment was only conducted once when the intervention finished, which made it impossible to adjust the treatment protocol during the intervention period. In addition, the primary outcome of this study was subjective and vulnerable to reporting bias. Objective measurements were also collected through the wrist-worn actigraphy. However, owing to the limited number of actigraphy devices, we only conducted 1 or 2 nights of actigraphy assessment, which might not accurately reflect the long-term sleep state.

## Conclusions

This randomized clinical trial found that 8 weeks of EA treatment was an effective and safe alternative therapy for treating insomnia in patients with depression. Our findings constitute subjective and objective evidence of the efficacy and safety of EA with standard care in treating comorbid depression and insomnia compared with SA with standard care or standard care alone. Further studies should focus on a longer treatment period with precise objective outcome assessment.

## Notes

Supplement 1.
Trial Protocol

Supplement 2. **eFigure.** Location of the Acupoints **eTable 1.** Seven Components of PSQI Changes **eTable 2.** Diary of Sedative Dose Change **eTable 3.** Adverse Events **eTable 4.** Blinding Result

Supplement 3.
Data Sharing Statement
