# Supplementary material for: Effect of Electroacupuncture on Insomnia in Patients With Depression: A Randomized Clinical Trial
Source: JAMA Netw Open. 2022 Jul 7;5(7):e2220563. doi: 10.1001/jamanetworkopen.2022.20563 (PMC9264041; doi:10.1001/jamanetworkopen.2022.20563)
Supplement: Supplement 1. — Trial Protocol [file jamanetwopen-e2220563-s001.pdf]

The efficacy and safety of electroacupuncture on treating depression related insomnia: a randomized controlled trial

Study Protocol

Shanghai Municipal Hospital of Traditional Chinese Medicine

Shanghai Mental Health Center

Changhai Hospital

1. Background

Depression and its related sleep disorders are becoming serious public health problems affecting people worldwide. The global point prevalence of MDD is 4.7%[1], and the estimation of a 12-month cumulative incidence of depression in China is 5.23%[2], causing an urgent need to improve depressive patients' health. Sleeping disorders including insomnia, hypersomnia and pavor nocturnus occur frequently in patients with depression [3]. Insomnia may occur in 60-80% of patients with major depressive disorders [4]; it is one of the most frequent residual symptoms of depression [5], and may persist even after depressive mood symptoms have been relieved [6].

Insomnia is characterized by persistent dissatisfaction with sleep quantity or quality for at least 4 weeks, with specific complaints of difficulty falling asleep, frequent nighttime awakenings, and/or awakening earlier in the morning than desired [7]. Insomnia may be triggered by different factors including psychiatric disorders, organic diseases and the intake of drugs or alcohol [8]. In fact, depressive symptoms are the largest and most consistent risk factors for insomnia because it affects the normal sleep-wake cycle [9 10]. Previous meta-analysis indicated moderate to large effect size (ES) improvement in depression as measured with the Hamilton Depression Rating Scale (ES = -1.29, 95%CI [-2.11, -0.47]), supporting that treating insomnia by Cognitive Behavioral Therapy for Insomnia (CBT-I) in patients with depression is effective and also have a positive effect on mood [11 12]. With regard to the current medical conditions in China, the need for CBT-I for patients with depression cannot be met. Although selective serotonin reuptake inhibitors (SSRIs) and barbiturates have considerably improved the efficacy and prognosis in the treatment of comorbid depression with insomnia, their side effects such as nausea, vomiting, tolerance, addiction, excessive sedation and neurological toxicity cannot be ignored [13-15]. What makes the pharmacotherapy more difficult is that some antidepressant drugs may worsen insomnia or cause daytime sleepiness [16], and high hypnotic dosages for insomnia is closely associated with worsened depressive outcomes [17]. In these cases, a drug-free alternative intervention is urgently needed as an effective and safe therapeutic approach for treating insomnia and depression.

Our previous study about acupuncture for primary insomnia demonstrated that acupuncture is an effective treatment to improve patients' sleep efficacy, prolong total sleep time and relieve patients' depressive mood[18]. The preliminary result of our pilot study [19] about the effect of electroacupuncture (EA) for depression related insomnia showed that the Pittsburgh Sleep Quality Index (PSQI) score in depression patients with electroacupuncture treatment obviously decreased (from 16.47 ± 1.89 to 9.83 ± 3.11), and there was significant difference between EA and sham EA ($p<0.01$). Meta-analysis also suggested that acupuncture combined with SSRIs is an effective and well-tolerated therapy for depression and adverse effects of antidepressants [20]. However, other studies showed that acupuncture is not significantly effective in relieving residual insomnia associated with depression [21 22]. As a result, randomized clinical trials in high quality are needed to evaluate the clinical effects and long-term effectiveness of acupuncture in the treatment of depression related insomnia.

2. Study aims

We planned this patient-blinded, multi-center, randomized and controlled trial with a sufficient observation period in three healthcare centers in Shanghai, China. We aim to observe the effects of EA treatment on sleep status, and eliminate the possible placebo effect by setting reasonable sham methods. The results will help to demonstrate if EA is an effective and safe therapy for improving sleep quality in patients with depression.

3. Design and Methods

3.1 Design

This is a multi-center, patient-assessor-blinded, randomized and controlled trial, aimed at evaluating the efficacy and safety of electroacupuncture for insomnia in depression patients and comparing the effects between electroacupuncture plus standard care, sham acupuncture plus standard care and simple standard care.

The trial will be performed in three healthcare centers in Shanghai: the acupuncture department in Shanghai Municipal Hospital of Traditional Chinese Medicine, the acupuncture department in Changhai Hospital of Shanghai and the therapeutic department in Shanghai Mental Health Center. We will recruit 270 patients who meet the inclusion criteria and randomly assign them to one of 3 groups, receiving electroacupuncture, sham acupuncture and/or standard medical care. After a week baseline, participants will enter an 8-month observation period in this trial. All treatments will be given 3 times a week (every other day) for 8 weeks. Participants will be assessed at the following time points: the baseline (1 week before treatment), the middle of the treatment (4 weeks after treatment starts), the end of the treatment (8 weeks after treatment starts) and follow-up (1 month, 3 months and 6 months after treatment finishes). All participants will complete the assessments by the PSQI, Actigraphy, HAMD, SAS and TESS (detailed trial process seen in Table 1). We will follow the Standards for Reporting Interventions in Clinical Trials of Acupuncture (STRICTA) [23] throughout the trial.

Table 1 Trial process chart

	**Baseline**	**Treatment period**			**Follow-up period**		
	Week -1	Week 0	Week 4	Week 8	Week 12	Week 20	Week 32
**Patients**							
Enrollment	×						
Signed informed		×					
consent	×						
Medical history	×						
Merger disease		×					
Randomization			×	×			
Intervention							
**Primary outcomes**	×		×	×	×	×	×
PSQI							
**Secondary outcomes**	×		×	×			
Actigraphy	×		×	×	×	×	×
HAMD	×		×	×			
SAS			×	×	×	×	×
TESS	×		×	×	×	×	×
Drug dose record			×	×	×	×	×
Patients' compliance							

3.2 Randomization and blinding

An online random allocation system will be designed by the central randomization system with a 1:1:1

ratio, using the Pocock and Simon minimization method 26. Staff of Shanghai BioGuider Medicinal Technology Co. Ltd (No. 2277 Zuchongzhi Road, Pudong New District, Shanghai) established the Data Analysis System (DAS) for the Electronic Data Capture (EDC) 5.0 system and prepared the randomization database. They offered technical support for the central randomization service and are not connected with the study. The system is based on the IIS (Internet Information Server) 5.0 as the Web Server, the SQL Server 2000 as the Database server and the ASP (Active Server Page) as the scripting language [24]. Central randomization has strict limits of authority; only researchers and the specialists from the Data and Safety Monitoring Board (DSMB) in this trial have access to the system. If the participant meets the inclusion criteria and agrees to join in the trial, a researcher who is not involved in the intervention in each healthcare center will login in to the central randomization system with his own username and password, enter the participant's personal information, and then get the randomized number and the group assignment. The patients' personal information will be protected and keep confidential to the acupuncturists and the assessors before, during and after the trial.

We will conduct a patient-assessor-blinded trial where participants are not aware of their group assignments and acupuncturists will not be involved in the outcome assessment or data analysis. Participants will be informed that they have an equal chance of allocation to the three groups. Participants who are assigned to the electroacupuncture (EA) or sham electroacupuncture (SA) will be treated in a closed unit to avoid communication. Furthermore, they will be asked to wear eye masks before and during the trial. Since there are inserted needles around participants' wrist joints, they will not be able to move their hands easily and cannot take off the eye masks. With these methods, participants will not be aware of the difference between EA and SA. To test the success of blinding, all participants in three centers will be asked by their acupuncturists whether they received EA or SA treatment at the end of treatment. Except the acupuncturists, other researchers including the statisticians, outcome assessors and data analysts are all blinded to the group assignments. All researchers will receive training on the specifications of this research method before the trial and strictly adhere to the task separation principle.

The flowchart of this trial is shown in Figure 1.

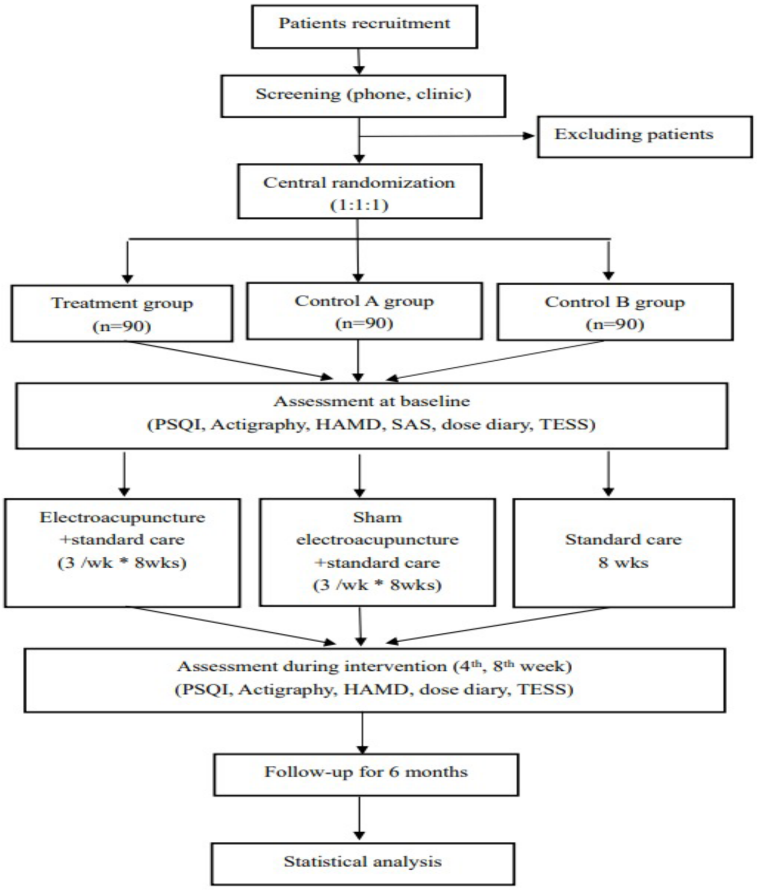

Figure 1

3.3 Patients

The study will include a total of 270 depression patients with insomnia. To ensure the precision of the results, we developed the following eligibility criteria.

3.3.1 Inclusion criteria

Participants meeting the following criteria will be included:

1. Male or female participants aged 18-70;
2. Participants who meet the diagnostic criteria of depression according to the Diagnostic and Statistical Manual of Mental Disorders, Fifth Edition (DSM-V) [25];
3. Participants whose HAMD score is 20-35 (mild to moderate depression);
4. Participants who have taken the same antidepressants for more than 4 weeks or have not taken antidepressants;
5. Participants who complained about insomnia during first screening;
6. Participants whose PSQI score is more than 7;
7. Participants who have not received acupuncture treatment for at least one year;
8. Participants who voluntarily agree with the investigation and sign a written informed consent form for the clinical trial.

3.3.2 Exclusion criteria

Participants who report any of the following conditions will be excluded:

1. Participants with secondary depressive disorders caused by organic diseases, medicine, or

psychotic disorders;

2. Participants who are in the depressive episode of bipolar disorder, or suffering from dysthymia, reactive depression and depressive syndrome caused by other diseases;
3. Participants who had severe diseases of cardiovascular or hematopoietic system, or had severe hepatic or renal insufficiency;
4. Participants with alcohol abuse or drug dependence;
5. Participants who refuse to wear the Actigraphy during the trial;
6. Pregnant or lactating women.

3.4 Recruitment

The participants will be recruited through hospital-based advertisements from outpatient clinics and from official websites of all three healthcare centers. If depression patients have interest in participating in the trial, they can take the phone screening first and then will be asked for face-to-face screening in any of the three healthcare centers where they need to fill in some forms with guidance from psychologists or doctors with professional training. Participants then will be asked to wear a wrist actigraphy to monitor their sleep quality for 3 days. Once the participants meet the inclusion criteria, they will be asked to sign the written informed consent form before intervention begins.

3.5 Sample size calculation

The sample calculation is based on changes in the primary outcome of this trial, the Pittsburgh Sleep Quality Index (PSQI) score. In our previous trial, we also used PSQI score as the primary outcome to evaluate and compare the effects between acupuncture, superficial acupuncture at sham points and sham acupuncture on treating depression related insomnia [26]. According to the preliminary results, the PSQI score of the acupuncture group at the end of the 8 weeks' intervention was 9.83±3.11 and that of the sham acupuncture group was 13.93±3.22. We assumed 0.2 of the PSQI difference is the superior effect.

H0: A-B<= Δ but H1: A-B> Δ

We used the following formula to calculate the sample size in this trial:

$N = \left[\frac{(Z_\alpha + Z_\beta)\,\sigma}{\delta - \Delta} \right]^2 \times 2$, where δ is the difference between group, Δ is the assumed superior effect threshold and N is the estimated sample size of each group. σ is the $[(S_1^2+S_2^2)/2]^{0.5}$

According to the previous study [27], the minimal clinically important difference (MCID) of PSQI is about 1.14-1.75. Since there will be a comparison between the Treatment group and the Control A group as well as a comparison between the Treatment group and the Control B group, a sample size of 27 in each group will have a power of 90% to detect the superior effect of 1.5 of PSQI at an α-value of 0.025 and a β-value of 0.1. Assuming a 10% dropout rate, a sample size of 30 for each group is needed. For a better power and quality control among centers, we decided the recruiting sample size to 30 for each group in each healthcare center. As a result, the total number of participants needed to be randomized is 270.

3.6 Intervention

Participants in Treatment group and Control A group will receive EA or SA treatment. Participants in these two groups will receive 24 sessions of different treatments, 3 times a week for 8 consecutive weeks. EA or SA treatment will be performed after skin cleansing, with patients wearing eye masks and lying supine. Each treatment will last for 30 minutes. The temperature of the treatment room cannot be lower than 25°C.

Considering the participants' psychological state, participants in all three groups can continue regular administration of antidepressants, sedatives, hypnotics or anxiolytics during the trial. They must record the dose, especially when they reduce the amount; and dose escalation will not be allowed unless the patient has consulted the psychiatrist. The patients will not be withdrawn the trial by changing the dose of the drug.

3.6.1 Treatment group

Participants in the Treatment group will receive electroacupuncture (EA) treatment. The acupuncture method of each acupoint is shown in Table 2. The regular acupuncture method will be applied at Baihui (GV20), Shenting (GV24), Yintang (GV29), bilateral Anmian (EX-HN22), Shenmen (HT7), Neiguan (PC6) and SanYinjiao (SP6). After needle insertion, rotating manipulation or lifting-thrusting manipulation will be applied for "Deqi" sensation. Two electrodes of the electro-stimulator (CMNS6-1, Wuxi Jiajian Medical Device CO., LTD, China) will be connected to the needles at Baihui (GV20) and Yintang (GV29) for 30 minutes, delivering a continuous wave. The frequency will be set to 30 Hz with a current intensity of 0.1 to 1 mA during the treatment, based on the tolerance of each patient.

3.6.2 Sham acupuncture group

Participants in the sham acupuncture group will receive sham electroacupuncture treatment at the same acupoints as the Treatment group. The acupuncture needles are produced by Asia-med GmbH&Co.KG (seen Figure 2), with the same appearance as those used in sham acupuncture treatment. The Streitberger Placebo needle[28,29] have been successfully used in our previous study [19,30]. When the tip of the blunt needles touches to the skin, the patient will get a pricking sensation but there is no real needle inserted into the skin. The electro-stimulator will be set beside the patients and two electrodes will be connected to the needles at Baihui (GV20) and Yintang (GV29). Acupuncturists will turn on the electro-stimulator, but all indicators will be set to "0". Participants will be informed when removing the needles after 30 minutes. Acupuncturists will use dry cotton balls to press the acupoints so that patients can feel the withdrawal of the 'needles'.

Table 2 Acupuncture method for each acupoint

**Acupoints**	**Needling method**	**Needle type**
**Baihui (GV20), Shenting (GV24)**	The angle between the needle tip and the scalp is 30°. Move the needle tip backward along the anterior-posterior midline, and then insert the needle for about 0.5-1cm.	0.25*25mm
**Yintang (GV29)**	Pinch the local skin, and then puncture obliquely for about 0.5-1cm.	0.25*25mm
**Shenmen (HT7)**	Puncture perpendicularly for about 0.5cm.	0.25*25mm
**Anmian (EX-HN22)**	The angle between the needle tip and the scalp is 30°. Puncture perpendicularly for about 1cm.	0.30*40mm
**Sanyinjiao (SP6), Neiguan (PC6)**	Puncture perpendicularly for about 1cm.	0.30*40mm

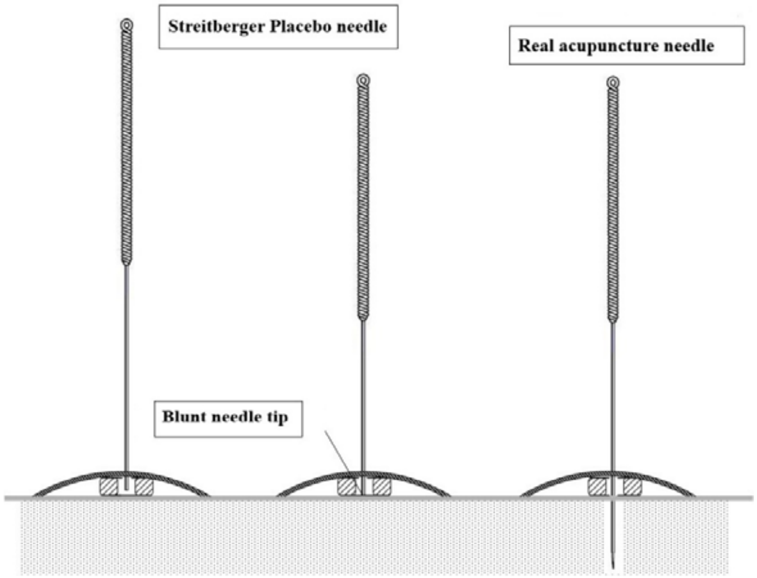

Figure 2

3.6.3 Control group

Standard care (also known as treatment-as-usual or routine care) in RCTs is frequently employed as the control condition to establish if the intervention is a significant improvement over existing practice [31]. In this trial, we set the control group as the standard care group to investigate the differences between EA treatment group and the blank control group so that the effects of EA for insomnia and depression will be observed more clearly. All 90 participants in three healthcare centers in the control group will continue taking in their routine antidepressants and/or sedative-hypnotics as before from baseline to 8 weeks. During the intervention period, if the patients have changes in their mental state and need help, the psychologists in Shanghai Mental Health Center will give professional advices and guidance. After finishing all the required scales and actigraphy records, they will get 10 sessions of free acupuncture treatment for insomnia.

3.7 Outcome Measurement

3.7.1 Primary outcome

The Pittsburgh Sleep Quality Index (PSQI) is a widely-used questionnaire with 19 items to assess sleep quality and disturbances over a one-month interval [32]. Four open-ended questions are followed by closed-ended questions that are rated on a 4-point Likert scale. The scores include the following indicators: subjective sleep quality, sleep latency, sleep duration, habitual sleep efficiency, sleep disturbances, use of medication, and daytime dysfunction. The accumulated scores of the seven indicators constitute the total score (ranging from 0–21). A higher score indicates worse sleep quality and more severe sleep disorders.

3.7.2 Secondary outcomes

1. The actigraphy (wActiSleep-BT. LLC, Pensacola, USA) worn on the patient's wrist can monitor the sleep quality, such as sleep onset, sleep latency, duration, awakenings during the night, etc. The software ActiLife6 (Version 6.8.1, ActiGraph, LLC) will be used to analyze every participant's sleep condition recorded in the actigraphy. The indicators used in our trial will be sleep efficiency (SE), sleep awakenings (SAW) and total sleep time (TST).

2. The Hamilton Rating Scale for Depression (HAMD) is an observer-rating questionnaire with 17 items used to assess the symptoms of patients diagnosed as suffering from depressive states [33]. Each item is rated in 3- or 5-point scales. A higher total score indicates a higher depression level.

3. The Self-rating Anxiety Scale (SAS) is primarily used as a measure of somatic symptoms associated with anxiety [34]. In using the scale, the participant will be asked to rate each item from 0-3 points according to how it applies to him or her within the past week. The standard score is the sum of the integer part of 1.25 times the raw score of the 20 items. A standard score of more than 50 points means the subject has anxious symptoms. A higher score indicates a more serious case of anxiety.

4. The dose dairy is a notebook where participants will be required to record their daily dose of antidepressants or sedative-hypnotics from baseline to 6 months follow-up, as well as the dosage time.

3.8 Adverse events

Any adverse events (described as unfavorable or unintended signs, symptoms or diseases occurring during the trial) related to the administration of antidepressant and sedative-hypnotics must be reported by patients and practitioners. These adverse events will be -recorded in the Treatment Emergent Symptom Scale (TESS) [35].

For the adverse events related to the acupuncture treatment, the most common ones include bleeding, faint, bruising ecchymoma, serious pain etc. These AE data will be assessed in terms of severity and causality, and the incidence will also be determined. The 3-point grading categories will be applied: grade 1, mild, grade 2, moderate, grade 3, severe or medically significant. The causality categories used will be certain, probable/likely, possible, unlikely, conditional/unclassified and unassessable/unclassifiable. The incidence of AEs was presented as the number of AEs per number of acupuncture sessions (%).

3.9 Statistical analysis

The statistical analyst will be blinded to the participants' personal information and their group assignment during the trial. The primary analysis will be a comparison of the changes of patients' PSQI score among three groups at 8 weeks after inclusion (comparison of the primary endpoint). The secondary analysis will be performed to assess the changes of the SE, TST and SA recorded in the actigraphy, as well as the HAMD scores and SAS scores from baseline to 8 weeks after inclusion. We will also count the number of patients who increase or decrease the drug dose, and then analyze the differences among three groups. All analyses will be performed on the intention-to-treat (ITT) population of participants who have at least one treatment. Missing data will be handled using the multiple imputation method, on the assumption that values at each time point follow a specific distribution calculated by the computer software R V.3.5. We will also perform a complete-case analysis without imputation of missing data, to find out if the results are consistent. Data analyses will be performed with the use of the statistical software SPSS V.20.0. The *t*-test will be used to compare the measurement data between either two groups from the baseline to 6 months follow-up; the rank sum test will be used for ranked data while the χ^2 test will be used to analyze categorical data. The significance level that will be used for statistical analysis with 2-tailed testing will be 2.5%. Data values will mainly be presented as Mean±SD.

3.10 Ethics and dissemination

All acupuncturists are licensed doctors with 3-5 years of experience in acupuncture treatment; and they will join in the clinical training before the intervention to ensure the standard real and sham

acupuncture operation in three centers. The trial has been approved by the Ethics Committee of Shanghai Municipal Hospital of Traditional Chinese Medicine, Shanghai, China (2017SHL-KY-04) and is registered with ClinicalTrials.gov (NCT03122080).

To guarantee the quality of the study, this trial will be carried out under the supervision of an independent DSMB. The DSMB consists of three experts from different fields: Professor Bingshun Wang in medical statistics from the School of medicine at Shanghai Jiaotong University, Dr. Lin Sun in psychology from the Department of Geriatrics at Shanghai Mental Health Center, and Professor Xueyong Shen in acupuncture from the Acupuncture College at Shanghai University of Traditional Chinese Medicine. The DSMB works to identify problems in the project, examine collected data, and control bias. Researchers in each healthcare center will promptly input data on the website (https://ecdm2.drugchina.net/crct2/) so that members in the DSMB can supervise the process at any time. Once they find problems or serious adverse events during the intervention, they can raise objections directly and even stop the trial until the problem has been resolved. Meanwhile, a qualified clinical trial expert (Lixing Lao) will be invited to monitor this study.

The results of this study will be published in peer-reviewed journals or presented at academic conferences.

4. Discussion

Acupuncture has been used to treat insomnia and some mental disorders since antiquity in China. According to the theory of traditional Chinese medicine, acupuncture provides balance to the body by stimulating specific acupoints, helping the body to achieve a state of relative equilibrium (the harmony of *"yin-yang")*, thereby restoring the normal sleep-wake cycle. Recent systematic reviews indicate that acupuncture could be an alternative therapy to medication for treating insomnia but needs further studies using large samples and a rigorous study design to confirm its role [36 37].

Previous RCTs always focus on either the acupuncture treatment for insomnia or that for depression, ignoring the relationship between these two diseases. Insomnia has been identified as the most common sleep disorder comorbid to depressive disorders [38]; so a reasonable acupuncture treatment program should be developed to normalize sleep disturbance and to relieve depressive mood as well. At the time of this writing, there are no similar RCTs about acupuncture for insomnia in depression patients that included a large sample size and were conducted in multiple healthcare centers. Our trial intends to present a strictly designed trial to study the effects of EA on insomnia in depression patients and to overcome some existing limitations, including illogical design, imperfect blinding method and practical difficulties in previous acupuncture clinical researches. With a long follow-up period, we will be able to explore the persistent effects of acupuncture for insomnia and determine for how long the therapeutic effect will last.

For patients in the EA group, we decided to use EA at Baihui (GV20) and Yintang (GV29), with the frequency set to 30 Hz during the treatment. According to the TCM theory, GV20 is the convergent point of six yang meridians as well as the foot Jueyin meridian; it is located on the top of the head, governs yang qi of the body and is the key point of calming mind. GV29 promotes the circulation of qi and blood in the head and restores the function of brain. EA at GV20 and GV29 enhances the effect of soothing nerves. In addition, a functional connectivity MRI (fcMRI) study suggested that EA at GV20 and GV29 may have effect on mental disorders[39]. Using fcMRI to identify the key cerebral functional region affected by EA at GV20 and GV29 found that the center of the cerebral network changed from the caudate nucleus to the parahippocampal gyrus and hypothalamus. The network centered on the

parahippocampal gyrus and hypothalamus primarily functioned in somatic movement, sensation, vision, hearing and language. This finding may indicate a mechanism for treating depression using EA at GV20 and GV29.

A frequency-specific neurochemical response in the central nervous system may be related to differential response of the body to low- and high-frequency EA stimulation and different peripheral and central pathways[40]. Previous research found that low frequency EA could be useful in clinical settings to manage pain [41] while high-frequency stimulation has more potent effects on 5-HT activity[42]. Thirty Hz separates the continuous wave of the electro-stimulator from disperse wave to dense wave and we chose 30Hz based on an acupuncture textbook [43].

Considering the complicated mental state of depression patients with insomnia, we will apply standard medication instead of unified antidepressants or sedative-hypnotics in this trial. Participants in all groups will continue taking in their individual routine dosage from baseline to 6 months follow-up. If their conditions obviously change during the study, they will be free to consult our psychologists from Shanghai Mental Health Center to adjust the dose. The use of standard care control groups has been the subject of much debate, with some pointing out that what constitutes standard care is unclear [44 45]. For better implementation of the standard care, researchers in our trial will try to carry out proper health education for all patients and supervise them in recording their daily medication dosage.

As a multi-center RCT conducted in a first-tier city, our study can provide more representative results about the role and value of acupuncture as a complementary and alternative therapy for insomnia and depressive moods than other single-center RCTs.

Considering the high prevalence of insomnia and depression in rural areas in China [46 47], the correlated heavy economic burden and serious public health problems cannot be underestimated. In future studies, the focus might be on the acupuncture treatment for insomnia in nationwide healthcare centers.

References

1. Ferrari AJ, Somerville AJ, Baxter AJ, et al. Global variation in the prevalence and incidence of major depressive disorder: a systematic review of the epidemiological literature. *Psychological Medicine* 2013;43(3):471-81.
2. Chin WY, Wan EY, Choi EP, et al. The 12-Month Incidence and Predictors of PHQ-9-Screened Depressive Symptoms in Chinese Primary Care Patients. *Annals of Family Medicine* 2016;14(1):47-53.
3. Luca A, Luca M, Calandra C. Sleep disorders and depression: brief review of the literature, case report, andnonpharmacologic interventions for depression. *Clinical Interventions in Aging* 2013;8(default):1033-38.
4. Winokur A, Gary KA, Rodner S, et al. Depression, sleep physiology, and antidepressant drugs. *Depression & Anxiety* 2001;14(1):19-28.
5. Nelson JC, Portera L, Leon AC. Residual symptoms in depressed patients after treatment with fluoxetine or reboxetine. *Journal of Clinical Psychiatry* 2005;66(11):1409-14.
6. Urrila AS, Karlsson L, Kiviruusu O, et al. Sleep complaints in adolescent depression: one year naturalistic follow-up study. *Bmc Psychiatry* 2014;14(1):283.
7. Medicine AAOS. International Classification of Sleep Disorders: Diagnostic and Coding Manual. *Chest* 2000;41(1):160-60.
8. Riemann D, Voderholzer U. Primary insomnia: a risk factor to develop depression? *Journal of*

Affective Disorders 2003;76(1-3):255.
9. MM O, T R. Place of chronic insomnia in the course of depressive and anxiety disorders. *J Psychiatr Res* 2003;37(1):9-15.
10. Ohayon MM, Caulet M, Lemoine P. Comorbidity of mental and insomnia disorders in the general population. *Comprehensive Psychiatry* 1998;39(4):185-97.
11. Gebara MA, Siripong N, DiNapoli EA, et al. Effect of insomnia treatments on depression: A systematic review and meta-analysis. *Depression and anxiety* 2018 doi: 10.1002/da.22776 [published Online First: 2018/05/22]
12. Trauer JM, Qian MY, Doyle JS, et al. Cognitive Behavioral Therapy for Chronic Insomnia: A Systematic Review and Meta-analysis. *Annals of Internal Medicine* 2013;47(2):191-204.
13. Wilson K, Mottram P. A comparison of side effects of selective serotonin reuptake inhibitors and tricyclic antidepressants in older depressed patients: a meta-analysis. *International Journal of Geriatric Psychiatry* 2004;19(8):754-62.
14. PRESKORN, Sheldon H. The Adverse Effect Profiles of the Selective Serotonin Reuptake Inhibitors: Relationship toIn VitroPharmacology. *Journal of Psychiatric Practice* 2000;6(3):153-57.
15. Chung KH, Li CY, Kuo SY, et al. Risk of psychiatric disorders in patients with chronic insomnia and sedative-hypnotic prescription: a nationwide population-based follow-up study. *Journal of Clinical Sleep Medicine Jcsm Official Publication of the American Academy of Sleep Medicine* 2015;11(5):543-51.
16. WA SM, PR B, PL C, et al. Sedative antidepressants and insomnia. *Revista Brasileira de Psiquiatria* 2011;33(1):91-5.
17. Li CT, Bai YM, Lee YC, et al. High dosage of hypnotics predicts subsequent sleep-related breathing disorders and is associated with worse outcomes for depression. *Sleep* 2014;37(4):803-09.
18. Yin X, Gou M, Xu J, et al. Efficacy and safety of acupuncture treatment on primary insomnia: a randomized controlled trial. *Sleep Medicine* 2017;37:193-200.
19. Yin X, Xu J, Dong B, et al. Efficacy and Safety of Electroacupuncture on Treating Depression Related Sleep Disorders: Study Protocol of a Randomized Controlled Trial. *Evidence-Based Complementray and Alternative Medicine,2016,(2016-12-29)* 2016;2016(3):1069597.
20. Chan YY, Lo WY, Yang SN, et al. The benefit of combined acupuncture and antidepressant medication for depression: A systematic review and meta-analysis. *J Affect Disord* 2015;176:106-17. doi: 10.1016/j.jad.2015.01.048 [published Online First: 2015/02/24]
21. Yeung WF, Chung KF, Tso KC, et al. Electroacupuncture for residual insomnia associated with major depressive disorder: a randomized controlled trial. *Sleep* 2011;34(6):807-15.
22. Chung KF, Yeung WF, Yu YM, et al. Acupuncture for residual insomnia associated with major depressive disorder: a placebo- and sham-controlled, subject- and assessor-blind, randomized trial. *The Journal of clinical psychiatry* 2015;76(6):e752.
23. Hugh MP, Altman DG, Richard H, et al. Revised STandards for Reporting Interventions in Clinical Trials of Acupuncture (STRICTA): Extending the CONSORT Statement. *Journal of Integrative Medicine* 2010;8(9):35-46.
24. Uher R, Payne JL, Pavlova B, et al. Major depressive disorder in DSM-5: implications for clinical practice and research of changes from DSM-IV. *Depression and anxiety* 2014;31(6):459-71. doi: 10.1002/da.22217 [published Online First: 2013/11/26]
25. Taoying L, Yan L, Jiyang P, et al. Study on Minimal Important Difference of the Pittsburgh Sleep

Quality Index Based on Clinical Trial of Traditional Chinese Medicine. *Journal of Guangzhou University of Traditional Chinese Medicine* 2013;30(4):574-78.

26. Pocock SJ, Simon R. Sequential treatment assignment with balancing for prognostic factors in the controlled clinical trial. *Biometrics* 1975;31(1):103.
27. Cai H, Xia J, Xu D, et al. A random allocation system using minimization method on Internet. *Journal of The Fourth Military Medical University* 2004;25(14):1278-80.
28. K S, J K. Introducing a placebo needle into acupuncture research. *Lancet (London, England)* 1998;352(9125):364.
29. Kleinhenz J, Streitberger K, Windeler J, et al. Randomised clinical trial comparing the effects of acupuncture and a newly designed placebo needle in rotator cuff tendinitis. *Pain* 1999;83(2):235-41. [published Online First: 1999/10/27]
30. Yin X, Gou M, Xu J, et al. Efficacy and safety of acupuncture treatment on primary insomnia: a randomized controlled trial. *Sleep Medicine* 2017
31. Freedland KE, Mohr DC, Davidson KW, et al. Usual and Unusual Care: Existing Practice Control Groups In Randomized Controlled Trials of Behavioral Interventions. *Psychosomatic Medicine* 2011;73(4):323-35.
32. Buysse DJ, Rd RC, Monk TH, et al. The Pittsburgh Sleep Quality Index: a new instrument for psychiatric practice and research. *Psychiatry Research* 1989;28(2):193-213.
33. Hamilton M. The Hamilton Rating Scale for Depression: Springer Berlin Heidelberg 1986.
34. Zung WWK. A Rating Instrument For Anxiety Disorders. *Psychosomatics* 1971;12(6):371-79. doi: 10.1016/s0033-3182(71)71479-0
35. Institute of Mental Health N. TESS (Treatment Emergent Symptom Scale-Write-in)1985.
36. Lee SH, Lim SM. Acupuncture for insomnia after stroke: a systematic review and meta-analysis. *Bmc Complementary & Alternative Medicine* 2016;16(1):228.
37. Dong B, Chen Z, Yin X, et al. The Efficacy of Acupuncture for Treating Depression-Related Insomnia Compared with a Control Group: A Systematic Review and Meta-Analysis. *BioMed Research International,2017,(2017-02-14)* 2017;2017(6):9614810.
38. Buysse DJ, Angst J, Gamma A, et al. Prevalence, course, and comorbidity of insomnia and depression in young adults. *Sleep* 2008;31(4):473-80.
39. Zhang G, Qu S, Zheng Y, et al. Key regions of the cerebral network are altered after electroacupuncture at the Baihui (GV20) and Yintang acupuncture points in healthy volunteers: an analysis based on resting fcMRI. *Acupuncture in medicine : journal of the British Medical Acupuncture Society* 2013;31(4):383-8. doi: 10.1136/acupmed-2012-010301 [published Online First: 2013/07/28]
40. Zhang ZJ, Wang XM, Mcalonan GM. Neural acupuncture unit: a new concept for interpreting effects and mechanisms of acupuncture. *Journal of Integrative Medicine* 2014;2012(3):429412.
41. Zhang R, Lao L, Ren K, et al. Mechanisms of acupuncture-electroacupuncture on persistent pain. *Anesthesiology* 2014;120(2):482-503.
42. Kwon Y-b, Kang M-s, Son S-s, et al. Different Frequencies of Electroacupuncture Modified the Cellular Activity of Serotonergic Neurons in Brainstem. *American Journal of Chinese Medicine* 2000;28(03n04):435-41.
43. Fang J. Acupuncture and Moxibustion Technique. Beijing: People's Medical Publishing House

2012:158.
44. Burns T. End of the road for treatment-as-usual studies? *British Journal of Psychiatry the Journal of Mental Science* 2009;195(1):5.
45. Liza D, Zarin DA, Emanuel EJ, et al. Considering Usual Medical Care in Clinical Trial Design. *PLoS Medicine,6,9(2009-9-29)* 2009;6(9):e1000111.
46. Zhou X, Bi B, Zheng L, et al. The prevalence and risk factors for depression symptoms in a rural Chinese sample population. *PloS one* 2014;9(6):e99692. doi: 10.1371/journal.pone.0099692
47. Dai J, Chiu HF, Xiang YT, et al. The prevalence of insomnia and its socio-demographic and clinical correlates in older adults in rural China: a pilot study. *Aging & Mental Health* 2013;17(6):761-5.
