# Supplementary material for: Effect of Electroacupuncture on Insomnia in Patients With Depression: A Randomized Clinical Trial
Source: JAMA Netw Open. 2022 Jul 7;5(7):e2220563. doi: 10.1001/jamanetworkopen.2022.20563 (PMC9264041; doi:10.1001/jamanetworkopen.2022.20563)
Supplement: Supplement 2. — eFigure. Location of the Acupoints eTable 1. Seven Components of PSQI Changes eTable 2. Diary of Sedative Dose Change eTable 3. Adverse Events eTable 4. Blinding Result [file jamanetwopen-e2220563-s002.pdf]

Supplementary Online Content

Yin X, Li W, Liang T, et al. Effect of electroacupuncture on insomnia in patients with depression: a randomized clinical trial. *JAMA Netw Open*. 2022;5(7):e2220563. doi:10.1001/jamanetworkopen.2022.20563

eFigure. Location of the Acupoints
eTable 1. Seven Components of PSQI Changes
eTable 2. Diary of Sedative Dose Change
eTable 3. Adverse Events
eTable 4. Blinding Result

This supplementary material has been provided by the authors to give readers additional information about their work.

© 2022 Yin X et al. *JAMA Network Open*.

eFigure. Location of the Acupoints

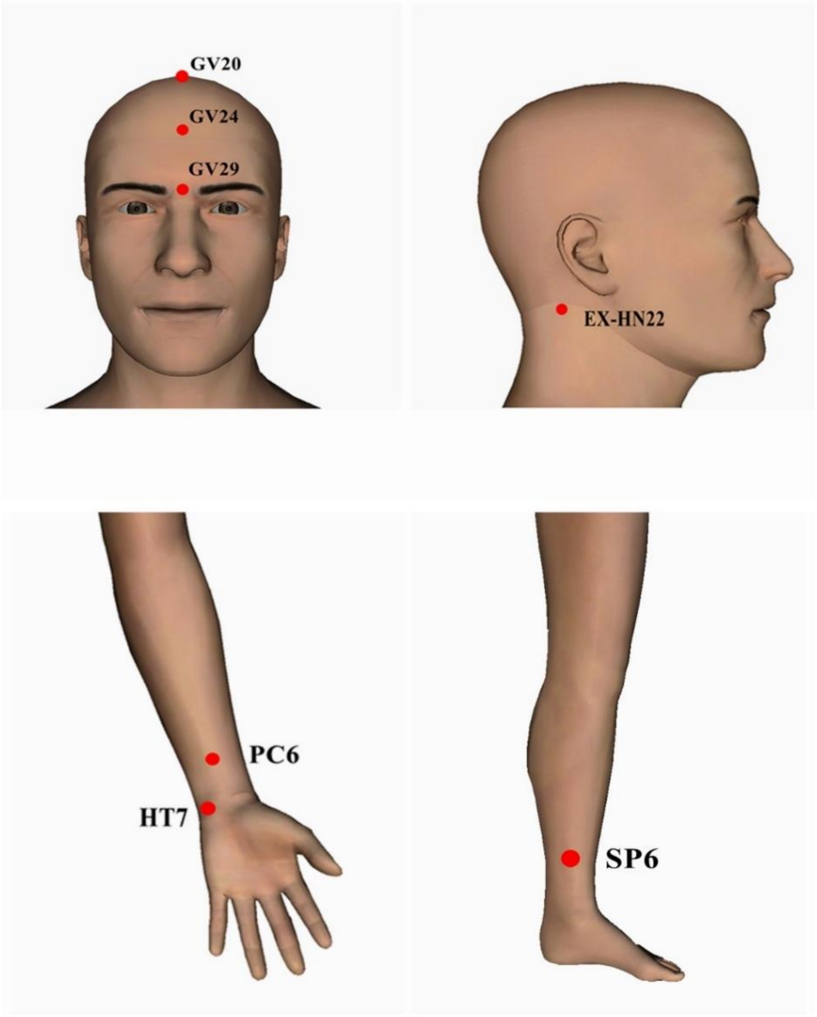

GV20: 5 cun superior to the anterior hairline, on the anterior midline of the head

GV24: 0.5 cun superior to the anterior hairline, on the anterior midline of the head

GV29: At the midpoint between the medial ends of eyebrows.

EX-HN22: At the midpoint between Yifeng (SJ17) and Fengchi (GB20).

PC6: 2 cun above of the transverse crease of the wrist, between the tendons of m. palmaris longus and m. flexor carpi radials

HT7: On the anterior and medial aspect of the wrist, radial to the flexor carpi ulnaris tendon at the palmar wrist crease.

SP6: On the tibial aspect of the leg, posterior to the medial border of the tibia and 3 cun superior to the prominence of the medial malleolus.

© 2022 Yin X et al. *JAMA Network Open*.

eTable 1. Seven Components of PSQI Changes

Outcomes	Mean PSQI scores, (SD)			EA vs. SA		EA vs. Control	
	EA group	SA group	Control group	Difference (95% CI)	P Value[a]	Difference (95% CI)	P Value[a]
**Sleep quality**							
**Week 4**	1.6 (0.7)	1.9 (0.7)	2.1 (0.8)	-0.3 (-0.4 to -0.1)	.004	-0.4 (-0.6 to -0.2)	<.001
**Week 8**	1.1 (0.6)	1.7 (0.6)	2.1 (0.7)	-0.7(-0.8 to -0.5)	<.001	-1.0 (-1.2 to -0.8)	<.001
**Sleep latency**							
**Week 4**	2.2 (0.9)	2.2 (1.0)	2.6 (0.8)	-0.2 (-0.4 to 0.0)	.15	-0.4 (-0.6 to -0.2)	<.001
**Week 8**	2.0 (0.9)	2.2 (0.9)	2.4 (0.8)	-0.4 (-0.6 to -0.1)	.004	-0.5 (-0.7 to -0.3)	<.001
**Sleep duration**							
**Week 4**	1.6 (1.0)	2.0 (1.0)	2.1 (1.0)	-0.4 (-0.6 to -0.2)	<.001	-0.5 (-0.7 to -0.3)	<.001
**Week 8**	1.2 (0.9)	1.8 (1.0)	2.0 (1.1)	-0.5 (-0.8 to -0.3)	<.001	-0.7 (-0.9 to -0.5)	<.001
**Habitual sleep efficiency**							
**Week 4**	1.8 (1.0)	1.8 (1.1)	1.9 (1.1)	-0.2 (-0.4 to 0.1)	.32	-0.2 (-0.4 to 0.0)	.05
**Week 8**	1.2 (0.9)	1.7 (1.1)	1.9 (1.0)	-0.6 (-0.9 to -0.4)	<.001	-0.8 (-1.0 to -0.5)	<.001
**Sleep disturbance**							
**Week 4**	1.4 (0.5)	1.7 (0.6)	1.8 (0.6)	-0.2 (-0.4 to -0.1)	.004	-0.3 (-0.5 to -0.2)	<.001
**Week 8**	1.2 (0.4)	1.6 (0.6)	1.7 (0.5)	-0.3 (-0.5 to -0.2)	<.001	-0.4 (-0.6 to -0.3)	<.001

© 2022 Yin X et al. *JAMA Network Open*.

**eTable 1 Seven Components of PSQI Changes (Continued)**							
**Use of sleeping medication**							
**Week 4**	1.6 (1.3)	1.9 (1.3)	2.3 (1.2)	-0.3 (-0.5 to -0.1)	.01	-0.4 (-0.6 to -0.2)	<.001
**Week 8**	1.2 (1.3)	1.8 (1.3)	2.3 (1.2)	-0.6 (-0.8 to -0.3)	<.001	-0.8 (-1.1 to -0.5)	<.001
**Daytime dysfunction**							
**Week 4**	1.5 (0.8)	1.8 (0.8)	1.9 (0.8)	-0.4 (-0.6 to -0.2)	<.001	-0.5 (-0.7 to -0.4)	<.001
**Week 8**	1.0 (0.8)	1.6 (0.7)	1.9 (0.9)	-0.6 (-0.8 to -0.4)	<.001	-0.9 (-1.1 to -0.7)	<.001

[a] P value by Bonferroni correction

Abbreviations: PSQI, Pittsburgh Sleep Quality Index; EA, Electroacupuncture; SA, Sham acupuncture.

© 2022 Yin X et al. *JAMA Network Open*.

eTable 2. Diary of Sedative Dose Change

	**EA group (n=15)** [a]		**SA group (n=18)**		**Control group (n=15)**	
	Deceased dose n (%)	Increased dose n (%)	Deceased dose n (%)	Increased dose n (%)	Deceased dose n (%)	Increased dose n (%)
**Wk0-Wk4**	10 (66.7)	0 (0.0)	9 (50.0)	4 (22.2)	4 (26.7)	4 (26.7)
**Wk4-Wk8**	3 (20.0)	1 (6.7)	5 (27.8)	6 (33.3)	5 (33.3)	3 (20.0)

[a] Rest patients kept their regular dose of sedatives.

© 2022 Yin X et al. *JAMA Network Open*.

eTable 3. Adverse Events

	EA group (n=90)	SA group (n=90)	Control group (n=90)
**AEs, n (%)**	7 (7.78)	4 (4.44)	1 (1.11)
**SAEs, n (%)**	0 (0)	0 (0)	0 (0)
**Treatment-emergent AEs, n (%)**	5 (5.56)	4 (4.44)	0 (0)
**Most frequent AEs**	Hematoma	Local pain	None

Abbreviations: AE: Adverse Events; SAE: Severe Adverse Events.

© 2022 Yin X et al. *JAMA Network Open*.

eTable 4. Blinding Result

Group	Guess (n, %) *			BANG's BI (95% CI)
	EA	SA	No idea	
**EA (n=90)**	62 (68.9%)	15 (16.7%)	13 (14.4%)	0.5 (0.4, 0.7)
**SA (n=90)**	56 (62.2%)	18 (11.1%)	16 (17.8%)	-0.4 (-0.6, -0.3)

* Percentages may not sum to 100 due to rounding.

Abbreviations: BI: Blinding Index

© 2022 Yin X et al. *JAMA Network Open*.
