# Supplementary material for: Effect of Electroacupuncture on Insomnia in Patients With Depression: A Randomized Clinical Trial
Source: JAMA Netw Open. 2022 Jul 7;5(7):e2220563. doi: 10.1001/jamanetworkopen.2022.20563 (PMC9264041; doi:10.1001/jamanetworkopen.2022.20563)
Supplement: Supplement 3. — Data Sharing Statement [file jamanetwopen-e2220563-s00.pdf]

Data Sharing Statement

Yin. Effect of Electroacupuncture on Insomnia in Patients With Depression. *JAMA Netw Open*. Published July 07, 2022. doi:10.1001/jamanetworkopen.2022.20563

Data
Data available: Yes
Data types: Deidentified participant data
How to access data: Send request email to Professor Shifen Xu: xu_teacher2006@126.com
When available: With publication

Supporting Documents
Document types: None

Additional Information
Who can access the data: Researchers whose proposed use of the data has been approved
Types of analyses: For a specified purpose
Mechanisms of data availability: After approval of a proposal, and with a signed data access agreement
